# Job Strain and Casual Blood Pressure Distribution: Looking beyond the Adjusted Mean and Taking Gender, Age, and Use of Antihypertensives into Account. Results from ELSA-Brasil

**DOI:** 10.3390/ijerph14040451

**Published:** 2017-04-22

**Authors:** Leidjaira Lopes Juvanhol, Enirtes Caetano Prates Melo, Marilia Sá Carvalho, Dóra Chor, José Geraldo Mill, Rosane Härter Griep

**Affiliations:** 1 National School of Public Health, Oswaldo Cruz Foundation, Rua Leopoldo Bulhões, 1480, Manguinhos, 21041-210 Rio de Janeiro, Rio de Janeiro, Brazil; leidjaira_lopes@hotmail.com (L.L.J); enirtes@fiocruz.br (E.C.P.M); dorachor@fiocruz.br (D.C.); 2 Scientific Computing Program, Oswaldo Cruz Foundation, Avenida Brasil, 4365, Manguinhos, 21040-360 Rio de Janeiro, Rio de Janeiro, Brazil; carvalho@fiocruz.br; 3 Department of Physiological Sciences, Federal University of Espírito Santo, Avenida Marechal Campos, 1468, Maruípe, 29042-755 Vitória, Espírito Santo, Brazil; jgmill@npd.ufes.br; 4 Laboratory of Health and Environment Education, Oswaldo Cruz Institute, Oswaldo Cruz Foundation, Avenida Brasil, 4365, Manguinhos, 21040-360 Rio de Janeiro, Rio de Janeiro, Brazil

**Keywords:** demand-control model, hypertension, job stress, method, psychosocial factor

## Abstract

Methodological issues are pointed to as the main sources of inconsistencies in studies about the association between job strain and blood pressure (BP)/hypertension. Our aim was to analyze the relationship between job strain and the whole BP distribution, as well as potential differences by gender, age, and use of antihypertensives. Additionally, we addressed issues relating to the operationalization of the exposure and outcome variables that influence the study of their inter-relations. We evaluated the baseline date of 12,038 participants enrolled in the Brazilian Longitudinal Study of Adult Health (ELSA-Brasil) (2008–2010), a multicenter cohort study of 35–74-year-old civil servants. Job strain was assessed by the Demand-Control-Support Questionnaire. The distribution of casual BP by categories of job strain was compared by a combination of exploratory techniques. Participants were classified into three subgroups (normotensives, medicated hypertensives, and unmedicated hypertensives), and analyses were stratified by gender and age. The relationship between job strain and casual BP varied along the whole outcome distribution. Hypertensive participants had greater differences in casual BP by job strain category, especially medicated hypertensives. Differences in casual BP were also greater for systolic than for diastolic BP and for older participants. No differences were encountered by gender. The exclusion of participants susceptible to misclassification for the exposure and outcome variables increased the differences observed between the categories of low and high job strain. In conclusion, the relationship between job strain and casual BP varied along the whole outcome distribution and by use of antihypertensive drugs, age, and BP parameter evaluated. Misclassification for exposure and outcome variables should be considered in analyses of this topic.

## 1. Introduction

Job strain resulting from the combination of high psychological demands from and low levels of control over the work process [1] is an important psychosocial risk factor associated with cardiovascular morbidity and mortality [2,3], and high blood pressure (BP) may be a mediator of that association [4]. Job strain can influence BP directly through neuroendocrine mechanisms, such as the activation of the hypothalamic-pituitary-adrenocortical axis and the sympathetic nervous system. This activation can cause alterations in vascular structure, which in turn can lead to hypertension [5,6,7]. Job strain may also have indirect effects on BP through habits such as smoking, drinking, unhealthy diet, and insufficient physical activity [8,9,10].

However, studies on the association between job strain and high BP, especially casual BP [11], have shown inconsistent results, with some studies reporting an association [4,12,13], others reporting no association [10,14,15], and some even suggesting an inverse association [16,17,18]. Some of these inconsistencies may be explained by methodological limitations, which may bias the true association between job strain and BP [19]. Indeed, in addition to the inherent complexity of measuring job strain and BP, results may be influenced by how these variables are operationalized [20]. Job strain is generally evaluated as a combination of scores for the dimensions of psychological job demands and job control, which are measured using two discrete scales. Job strain is then classified into in four job types (or quadrants): low strain (low demand and high control); active (high demand and high control); passive (low demand and high control); and high strain (high demand and low control) [1]. Generally speaking, the mean or quantiles of the study population are used as the cut-off to classify individuals into categories of high/low psychological job demands and high/low job control [21], but this procedure may not be appropriate when the study population is insufficiently heterogeneous.

BP is a cardiovascular risk factor that displays a continuous distribution [22], but it is analyzed as a binary variable in most studies [4,23]. Generally, individuals are dichotomized into normotensives and hypertensives in order to simplify the statistical analyses and to make it possible to calculate measures of association such as odds ratios and relative risks, which are easier to interpret [24]. However, dividing this continuum of risk based on an arbitrary cut-off point entails limitations: (1) it can cause information loss, which means reduced statistical power to detect a true association [25]; (2) the information contained within each category is not evaluated because individuals belonging to the same stratum are considered homogeneous, even though they may be substantially different [26]; (3) at values close to the cut-off point, similar individuals are classified into different strata [27]; and (4) classifying individuals as normotensive and hypertensive eliminates any opportunity to evaluate systolic BP (SBP) and diastolic BP (DBP) separately.

The variation in the results of studies on job strain and high BP may also stem from the fact that the effects of job strain on BP are heterogeneous within a population. In addition to differences by gender [11], more pronounced associations have been observed among people with low socioeconomic status [28], those with low social support [29], among men who are manual workers, men with a lower education level, and older men [30]. Thus, different factors seem to interact, contributing to the complexity of the relationship.

Clearly, exploring the relationship between job strain and BP is a complex, but extremely important task, as high BP is the main risk factor for the global burden of disease [31]. Despite the considerable volume of publications on the subject [4,11], few studies have reflected on new methodological approaches that might help us to understand this relationship.

Therefore, the goal of the present paper was to analyze the relationship between job strain and casual BP by exploring the whole BP distribution and to investigate potential differences by gender, age, and use of antihypertensives. Additionally, we addressed methodological issues relating to the operationalization of the exposure and outcome variables that influence the study of their interrelation.

## 2. Materials and Methods

### 2.1. Study Population

This study uses baseline data from the Brazilian Longitudinal Study of Adult Health (ELSA-Brasil), a multicenter cohort study conducted in higher education and research institutions in six state capitals of Brazil (Federal Universities of Bahia, Espírito Santo, Minas Gerais, Rio Grande do Sul, University of São Paulo, and the Oswaldo Cruz Foundation). All current or retired employees of these institutions, of both sexes, aged 35–74 years old were eligible for the study. Efforts were made to meet recruitment goals by gender, age, and occupational category so as to ensure a wide socioeconomic gradient across the study population. Participants were recruited through various media outlets (on-site and radio announcements, mailings, and outdoor billboards) and active recruitment (from a randomly-ordered list of employees). Of the 16,435 people who expressed interest in participation, 15,105 completed the baseline examination (2008–2010). This examination consisted of an interview, a clinical evaluation, and laboratory tests, which were performed in the study clinic of the participant’s institution. More details about the study design and recruitment methods can be found elsewhere [32,33,34].

For the present analysis, we excluded all retired participants and participants without complete information on the variables analyzed, leaving us with a study sample of 12,038 participants. Occupation in the study sample varied widely and included unskilled manual workers, technical and clerical workers, and professionals such as health care workers, university faculty, and researchers. The research protocol of ELSA-Brasil was approved by the research ethics committees of each of the participating institutions (São Paulo University, CEP-HU 659/06; Oswaldo Cruz Foundation, CEP-Fiocruz 343/06; Bahia Federal University, CEP-ISC 027/06; Minas Gerais Federal University, COEP 186/06; Espírito Santo Federal University, CEP-CCS 041/06; and Rio Grande do Sul Federal University, CEP-HCPA 194/06) and by the National Research Ethics Committee (CONEP 13065). Informed consent was obtained from all study participants.

### 2.2. Study Variables

#### 2.2.1. Job Strain

Job strain was measured by the Brazilian version [35] of the Swedish Demand-Control-Support Questionnaire [36], which has demonstrated satisfactory reproducibility and internal consistency [35], and the dimensional structure of which has been evaluated [37,38]. The level of psychological job demands was determined by five items relating to work load, the pace of the activities involved, and the difficulty in performing them. Level of job control was determined by six items relating to the degree of decision-making autonomy and use of intellectual skills. The scores obtained for psychological job demands (5–20 points) and job control (6–24 points) were dichotomized from the medians of these dimensions (14 and 18 points, respectively), and, based on these, job strain was categorized according to the quadrant formulation proposed by Karasek [1]: low strain (low demand/high control); active (high demand/high control); passive (low demand/low control); and high strain (high demand/low control).

#### 2.2.2. Casual Blood Pressure

BP was measured at the study clinic in a temperature controlled room (20–24 °C); measurements were taken in the morning at the beginning of the clinic visit and before blood sample collection. An overnight fast (10 to 14 h) was required for glucose tests, which contributed to standardizing the BP measurements in relation to food intake. Three consecutive BP measurements were taken from the left arm at 1-min intervals using a validated oscillometric device (Omron HEM 705CPINT, Omron Co., Kyoto, Japan), with participants in a seated position with an empty bladder and after a 5-min rest. Casual BP was considered to be the mean of the last two measurements [39].

#### 2.2.3. Blood Pressure-Related Variables

Participants were asked about any continuous drug use in the two weeks prior to the baseline examination, and antihypertensive drugs were characterized according to their pharmaceutical classification. Participants were considered as antihypertensive drug users if they reported antihypertensive drug use and responded affirmatively to the question: ‘Were any of the drugs you took in the past two weeks for hypertension (high blood pressure)?’ Hypertension was defined as SBP ≥ 140 mmHg or DBP ≥ 90 mmHg or use of antihypertensive drugs.

Based on the information above, participants were classified into three subgroups: normotensives, medicated hypertensives, and unmedicated hypertensives. We did not take into account the use of other drugs that might interfere with BP but are not classified as antihypertensive drugs, such as tranquilizers and antidepressants. A self-reported medical diagnosis of hypertension was used only as additional information to characterize the study population by casual BP levels and was not considered in classifying hypertension.

#### 2.2.4. Covariables

Age in years was considered as a continuous and categorical variable (<50 years and ≥50 years). Participants reported their race/color using the categories from Brazil’s population census; Black, Brown, White, Asian, or Indigenous. The other covariables considered were gender (male and female) and education level (less than high school, complete high school, college, and postgraduate).

### 2.3. Statistical Analysis

Initially, the distribution of the exposure and outcome variables were evaluated in entire the study sample using the distribution of the dimension scores by job strain quadrant and the means of casual BP by BP-related variables. After this, the job strain variable was dichotomized into two job strain categories; high job strain (high strain and passive quadrants) and low job strain (low strain and active quadrants). This was possible because the distribution of casual BP was quite similar in the combined quadrants. The distribution of casual BP by job strain category was compared visually by constructing kernel density graphs. Then two-sample Kolmogorov-Smirnov (K-S) tests were performed to test the statistical significance of the differences observed between the casual BP distributions. Additionally, the differences in the means of casual BP between the high and low job strain categories, the respective 95% confidence intervals (CI), and *t*-tests for difference in means were calculated. Finally, in order to identify the magnitude and location of the differences found along the whole casual BP distribution, the distributions were compared using quantile-quantile (Q-Q) plots. Casual BP distribution quantiles (1st–19th quantile) for the high job strain category (*y*-axis) were plotted against the quantiles for the low job strain category (*x*-axis), and deviations from the straight line *x* = *y* were examined. All analyses were conducted among the three subgroups (normotensives, medicated hypertensives, and unmedicated hypertensives), and SBP and DBP were evaluated separately. The analyses were also stratified by gender and age. All analyses were performed using R 3.1.2, [40] and graphical outputs were obtained using a ggplot2 package [41].

## 3. Results

### 3.1. Job Strain

**Figure 1:**
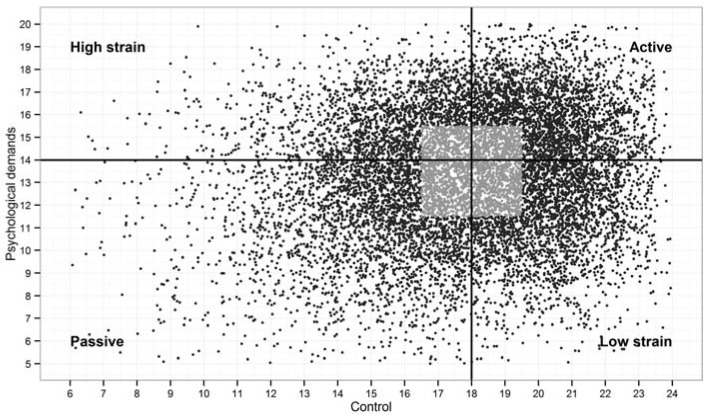
Distribution of participants by psychological demands and control dimension scores and Karasek quadrants in the Brazilian Longitudinal Study of Adult Health (ELSA-Brasil), 2008–2010 (*N* = 12,038). The black lines perpendicular to the *x* and *y* axes represent the medians observed in the study sample for the dimensions of job control and psychological job demands, respectively. The grayed area in the center of the graph refers to the exclusions made for subsequent analyses.

The study sample displayed a homogeneous distribution of scores for the dimensions of psychological job demands and job control. Considering the possible range of scores for these two dimensions (5–20 and 6–24, respectively), the distribution was concentrated at relatively high scores for psychological job demands and at high scores for job control. As the quadrants were formed using the median of the distribution for the study sample, individuals grouped in different quadrants were quite similar in terms of their scores for the dimensions of the demand-control model (Figure 1).

Because of this homogeneity, and in order to better discriminate between the job strain quadrants, participants with scores close to the mean of the study sample, i.e., those that fell within the second tertile for both psychological job demands and job control (*n* = 2353), were excluded to reduce the likelihood of misclassification [42]. This portion of the study population (approximately 20% of the total) is represented by the grayed area in the central portion of the distribution (Figure 1).

### 3.2. Casual Blood Pressure

**Figure 2:**
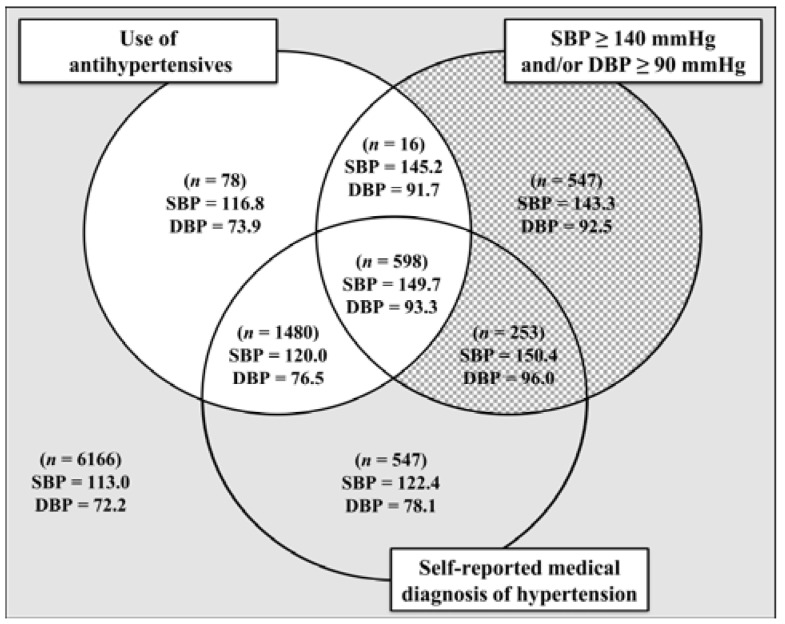
Casual blood pressure means and distribution for the three subgroups (normotensives [grayed area], medicated hypertensives [whitened area], and unmedicated hypertensives [checkered area]) by the blood pressure-related variables (use of antihypertensive drugs, SBP ≥ 140 mmHg and/or DBP ≥ 90 mmHg, and self-reported hypertension) in ELSA-Brasil, 2008–2010 (*n* = 9685). DBP = diastolic blood pressure; SBP = systolic blood pressure.

The distribution of the remaining study sample (*n* = 9685) was then evaluated by the BP-related variables (Figure 2). Different casual BP means were observed for the various combinations of these BP-related variables. Among participants initially classified as normotensive (grayed area), those who reported a medical diagnosis of hypertension displayed substantially higher mean BPs (122.4/78.1 mmHg, as compared with 113.0/72.2 mmHg among normotensives who did not report a medical diagnosis of hypertension). These individuals may have displayed higher BPs a few times in their lives and received a diagnosis of hypertension based on this. However, as they returned to borderline values, they may have been using only non-pharmaceutical treatment to control their BP.

Therefore, in order to reduce the likelihood of misclassification of hypertension, we excluded 547 individuals who were initially classified as normotensive (grayed area) but reported a medical diagnosis of hypertension. We further removed 115 participants who were initially classified as normotensive (grayed area) or unmedicated hypertensive (checkered area) but reported using antihypertensive drugs for other purposes, as they might skew the estimates. This left us with a sample after the exclusion of those susceptible to misclassification of 9023 participants.

### 3.3. Relationship between Job Strain and Casual Blood Pressure Distribution

In our original study sample of 12,038 participants, the difference in casual BP means between the low and high job strain categories was 2.41 mmHg (95% CI: 1.83, 3.00) for SBP and 1.30 mmHg (95% CI: 0.92, 1.69) for DBP. After the exclusion of those susceptible to misclassification (*n* = 3015 participants), these values were 2.71 mmHg (95% CI: 2.02, 3.39) and 1.46 mmHg (95% CI: 1.01, 1.91), respectively, which represents an increase in the differences of 0.30 mmHg for SBP and 0.16 mmHg for DBP. Of these 9023 individuals remaining in our study sample (6066 normotensives, 2172 medicated hypertensives, and 785 unmedicated hypertensives), 4653 (51.6%) were classified as having high job strain, 52.1% were women, and 51.4% declared themselves White, 29.4% Brown, 15.9% Black, 2.4% Asian, and 0.9% Indigenous. The mean age was 49.3 years (standard deviation = 7.4 years; variation = 35–72 years), and 47.6% were aged ≥50 years. Over one-third (38.6%) had postgraduate education, 15.1% had higher education, and 11.3% had not completed high school (Table 1).

**Figure 3:**
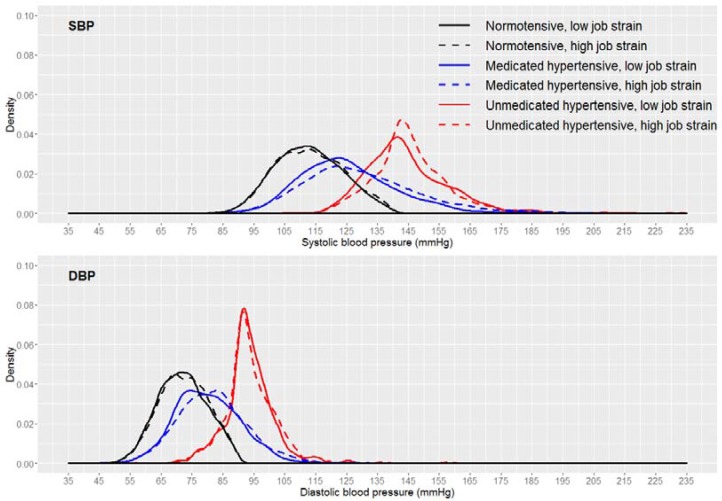
Casual blood pressure distribution by job strain category, considering the three subgroups (normotensives, medicated hypertensives, and unmedicated hypertensives) in ELSA-Brasil, 2008–2010 (*n* = 9023).

As expected, casual BP among normotensives was lower than among hypertensives, while medicated hypertensives occupied an intermediate position (Figure 3). Furthermore, for SBP, the distribution curves for the high and low job strain categories were overlaid only for normotensives. Among medicated and unmedicated hypertensives, there was a separation between the curves. For DBP, however, the overlay occurred for all three subgroups (normotensives, medicated hypertensives, and unmedicated hypertensives).

**Figure 4:**
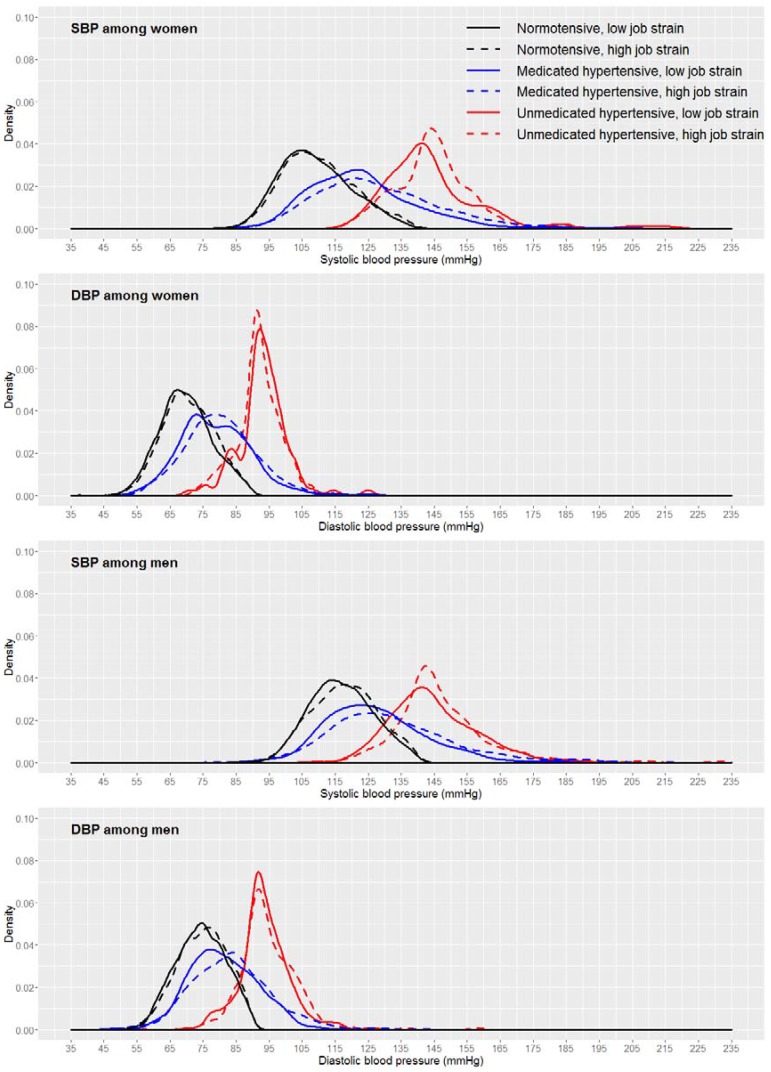
Casual blood pressure distribution by job strain category, considering the three subgroups (normotensives, medicated hypertensives, and unmedicated hypertensives), stratified by gender in ELSA-Brasil, 2008–2010 (*n* = 9023).

The distribution curves of normotensives, medicated hypertensives, and unmedicated hypertensives for SBP and DBP by job strain category were also evaluated by gender (Figure 4). The distribution pattern of casual BP for both genders was similar. However, the distances between the low and high job strain curves for SBP were less marked among men, except for normotensives.

**Figure 5:**
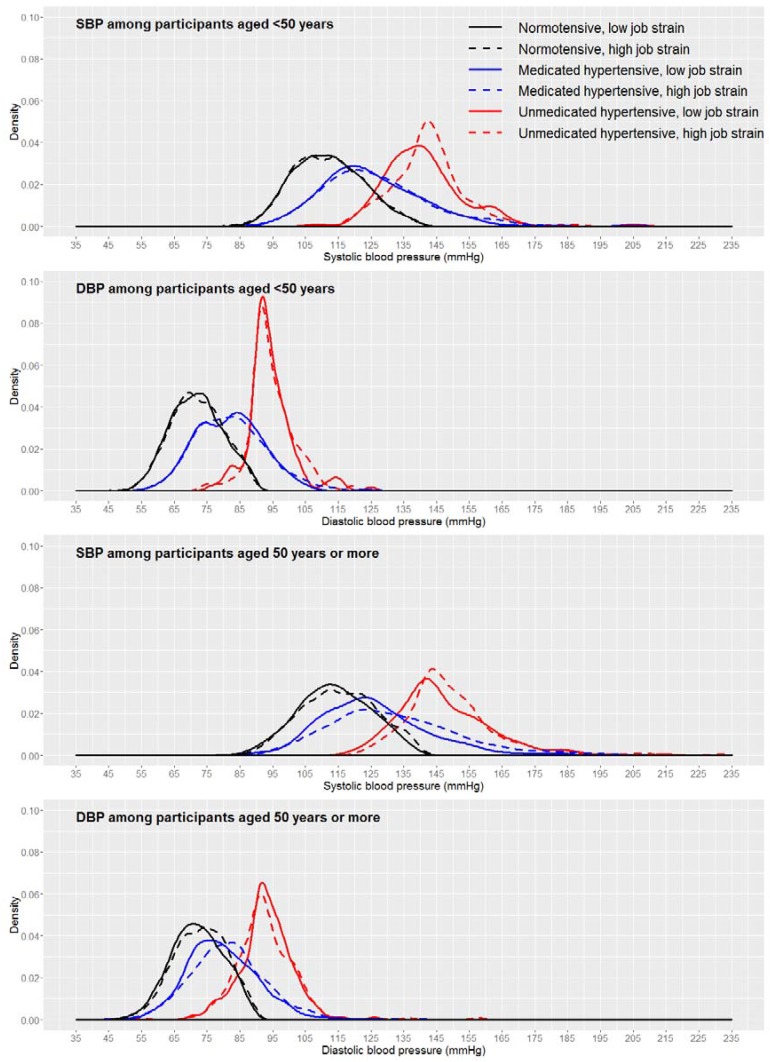
Casual blood pressure distribution by job strain category, considering the three subgroups (normotensives, medicated hypertensives, and unmedicated hypertensives), stratified by age in ELSA-Brasil, 2008–2010 (*n* = 9023).

When the same curves were stratified by age (Figure 5), the distributions of DBP by job strain category continued to be overlaid, regardless of subgroup (normotensives, medicated hypertensives, and unmedicated hypertensives) or age. For SBP, however, differences were observed. Among participants aged <50 years, high job strain produced displacement to the right only among unmedicated hypertensives. This did not occur among those aged ≥50 years, among whom the distances between the curves were similar for medicated and unmedicated hypertensives, and was less pronounced among the normotensives.

The two-sample K-S tests indicated a similar pattern to that observed in the kernel density graphs (Table 2). In the total study sample, the differences between the SBP distributions for the low and high job strain categories were larger and significant for medicated and unmedicated hypertensives. A similar pattern was observed by gender. However, as observed in the density curves, the differences between the low and high job strain categories were slightly greater among medicated and unmedicated hypertensive women. In the analysis by age, no significant difference between the comparison groups was observed among participants aged <50 years. On the other hand, among those aged ≥50 years, significant differences were observed among normotensive, medicated, and unmedicated hypertensive participants, and they were greater and similar for both subgroups of hypertensives. For DBP, lower but significant differences were also observed, especially among medicated hypertensives, except medicated hypertensives aged <50 years. Table 2 also shows the differences in casual BP means between the low and high job strain categories. For SBP, the greatest difference between means was observed among medicated hypertensives, except in those aged <50 years. For DBP, the differences between means were also generally greater among medicated hypertensives. These differences were also greater for SBP than DBP, among men, and among those aged ≥50 years.

**Figure 6:**
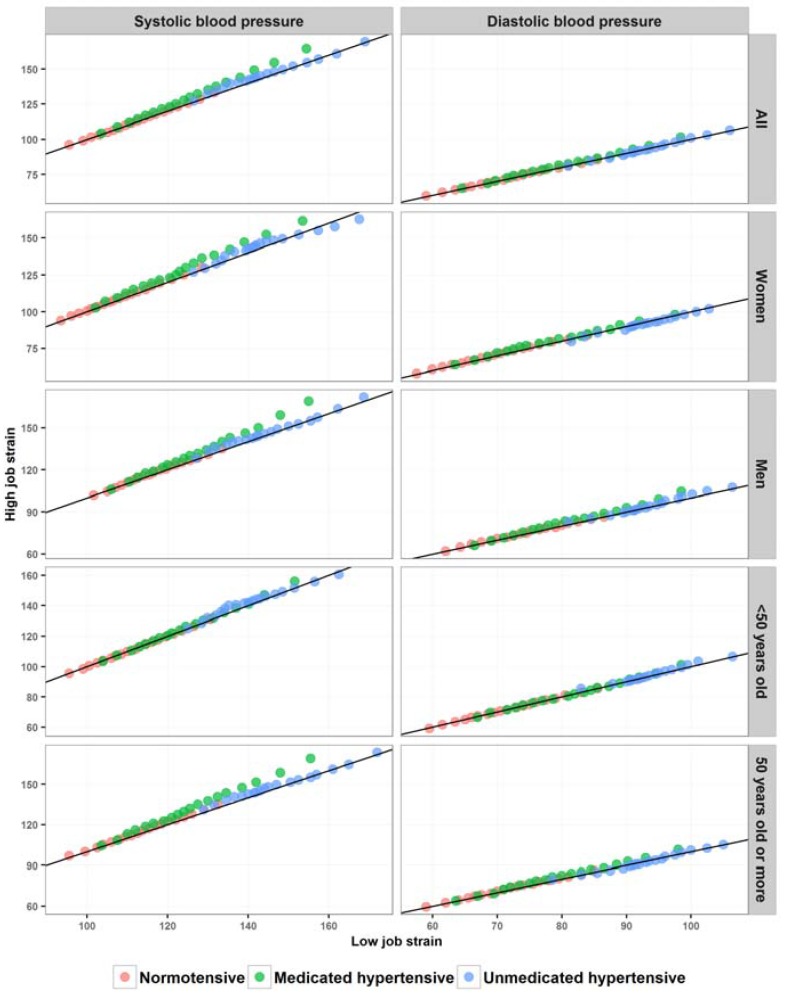
Quantile-quantile plot comparing casual blood pressure distribution by job strain category, considering the three subgroups (normotensives, medicated hypertensives, and unmedicated hypertensives), for the total study sample and stratified by gender and age in ELSA-Brasil, 2008–2010 (*n* = 9023). Displacements from the line *x* = *y* (black line) indicate differences between the quantiles of the two groups. Displacement upwards indicates that the value of the quantile in question is greater in the high job strain category; displacement below the line indicates that the value of the quantile in question is greater in the low job strain category.

Differences between the quantiles of the high and low job strain categories varied along the whole casual BP distribution (Figure 6). Most of the displacements (deviations from the straight line *x* = *y*) were above the straight line, indicating a higher casual BP among those with high job strain. For SBP, the differences were greater among medicated hypertensives. Among those aged <50 years, however, the displacements were slightly greater among unmedicated hypertensives. The displacements were also similar between men and women and greater among those aged ≥50 years. When compared by job strain category, the SBP distributions among normotensives and the DBP distributions in the three subgroups (normotensives, medicated hypertensives, and unmedicated hypertensives) were quite similar, regardless of gender or age.

## 4. Discussion

Our results show that the relationship between job strain and casual BP is heterogeneous in our study sample and that new strategies for analyzing this relationship may reveal similar situations in other populations. In spite of the importance of the usual multiple models, in which the coefficient estimated represents only the mean effect after adjustment for all other covariates, we decided to use an entirely different approach. This field of research is replete of association studies on job strain and BP/hypertension, including regression models adjusted and even over-adjusted for mediators and colliders and interaction analysis. Instead, we aimed to look beyond the adjusted mean by exploring the relationship between job strain and the whole BP distribution, taking gender, age, and use of antihypertensives into account. Our purpose was to understand how the exposure and outcome variables are distributed and how this distribution affects the results. Multiple models, although they can be easily fitted, do not permit the insights obtained with the strategy applied here.

We ascertained that density curves are a useful tool that can enable the comparison of BP distributions, although this comparison is only visual and there is no uncertainty. Even when one distribution is displaced in relation to another, a comparison based on the area under the curve does not express the phenomenon, because the area under the curve is always equal to one. The two-sample K-S test is a useful nonparametric method, which checks whether two datasets have different distributions and attempts to handle uncertainty. However, as it only considers the largest distance between the two empirical distribution functions, information about the magnitude and location of the differences along the distributions is not provided. Additionally, it is influenced by the sample size [43]. The use of means, on the other hand, also has its limitations, because, in addition to being measures that are very sensitive to extreme values, the differences between means and their 95% CIs are taken to indicate real differences between the distributions only under conditions of normality. In addition, when the distributions are asymmetrical, as in our study, comparing only the means may obscure important aspects of the relationship under study. Two distributions may be similar in form and have different means or they may have similar means and differ in specific quantiles [44]. Thus, comparison of casual BP levels by distribution quantiles was the most useful option, because, in addition to being robust measures, they make it possible to quantify and locate differences across the whole extent of the distribution.

The greatest differences in the distributions of casual BP by job strain category were found among hypertensive participants, suggesting a greater susceptibility to the adverse effects of job strain in this group. We hypothesized that, among hypertensives, casual BP responds more markedly to job strain because the unfavorable biological and psychosocial substrate that fostered the sustained increase in BP in this group interacts positively with job strain. Corroborating this finding, a study of young people found no overall association between job strain and work site BP, although a significant association was observed among those diagnosed as hypertensive [45]. Positive interaction has also been observed between cardiovascular hyperreactivity and stress and psychological job demands in the progression of atherosclerosis [46], and a more marked cardiovascular response to behavioral stress among individuals with suboptimal BP has also been observed [47].

In epidemiological studies, investigations of associations among diverse exposures and outcomes have evolved in recognition of the complexity of such relations [48]. The relationship between job strain and BP is also complex. Schwartz et al. [49] found that workplace stressors interact with psychological characteristics to produce a given level of perceived stress, and the magnitude of any subsequent raise in BP depends on the intensity of the reaction from the cardiovascular system. Accordingly, the coexistence of a genetic substrate and different psychosocial stressors serves to heighten certain individuals’ susceptibility to the adverse effects of job strain [50,51]. This appears to be particularly important among hypertensives, as the presence of an unfavorable biological and psychological substrate may have favored the sustained increase in BP in this group.

Although not observed in our research, various studies have signaled important gender differences in the relationship between job strain and BP, with the findings being less consistent for women [11,13,29]. On the other hand, our results did point to greater differences between casual BP distribution by job strain category among medicated hypertensives. In our study population, which has a high overall education level [34] and a high awareness of the condition of hypertension and use of antihypertensive drugs (80.2% and 76.8%, respectively) [52], this finding may indicate the presence of more severe hypertension in medicated hypertensives, as compared with unmedicated hypertensives. This result is consistent with the greater differences in casual BP by job strain category observed among more elderly participants, who characteristically displayed more severe hypertension that was more difficult to control [53,54]. Meanwhile, among younger participants, the use of antihypertensive drugs appeared to attenuate the adverse effects of job strain, perhaps because hypertension is easier to control in this group. However, our findings need to be confirmed in further studies in which BP is measured at more than one period of the day, such as at work and during sleep.

Our findings showed that the study of the relationship between job strain and casual BP is influenced by methodological issues surrounding the definition of these exposure and outcome variables. Non-differential misclassification is one of the most common and important sources of bias in this field, which leads to an underestimation of the adverse effects of job strain on BP [11]. In line with this, our results did indicate a reasonable increase in differences between the comparison groups after removing individuals susceptible to misclassification for job strain and BP-related variables. Thus, how study variables are analyzed does seem to influence results, which may explain some of the inconsistencies encountered in previous studies on the association between job strain and BP.

Job strain is one example of a situation in which the variables evaluated are by nature categorical but constitute theoretical constructs that can be measured using multidimensional instruments based on discrete scales. Although job strain can be formulated in different ways [42,55], the procedure most commonly used is classification by quadrants [20,21,56], which can have important implications. As population cut-off points classifying individuals by levels of psychological demands and control have not been developed for most countries, most studies use the mean or quantiles (median, tertile, or quartile) of the study sample to determine cut-offs [21]. In samples that are occupationally more homogeneous, levels of psychological job demands and job control are also more similar, which makes it difficult to form sufficiently different comparison groups and can lead to an underestimation of measures of association [11]. This implication is particularly important in studies of job-related issues, because many such studies target specific groups of workers who share some characteristics [57,58,59]. Given these constraints, we excluded the segment of the study sample located in the central portion of the distribution, which is more prone to misclassification [42]. As a result, the job strain quadrants became much better discriminated. Although this technique is not often used in the literature, it is very useful when variables are operationalized using quadrants.

In our analyses of the relationship between job strain and casual BP, we combined the quadrants into two groups; low job strain (low strain and active quadrants) and high job strain (high strain and passive quadrants). We used this strategy because preliminary analysis found that BP distributions were quite similar among the combined groups and that their distribution curves were overlaid. Thus, adopting this procedure was a posteriori decision based on our results that aimed to improve clarity in presenting and interpreting the results. In this study, we did not intend to evaluate job control or job demands separately. However, based on the similarity among the combined groups, we hypothesize that job control may be a more important predictor of BP status than job demands in our study sample, which is in line with what has been reported by other studies of cardiovascular risk [3,60]. We aim to test this hypothesis in future analyses.

We aimed to evaluate the original concept of job strain proposed by Karasek [1], which does not include the dimension of social support at work added later by Johnson and Hall [61]. A Brazilian study that evaluated the dimensional structure of the demand-control-support questionnaire showed that the best model fit was achieved by removing the dimension of social support at work [38]. Moreover, in our preliminary analysis, scores in this dimension displayed high levels and low variability. The reason may be that the ELSA-Brasil participants are civil servants, i.e., they have permanent jobs, which may contribute to reduced competitiveness and increased social support among co-workers. Thus, despite its role as a potential effect modifier in the association between job strain and health outcomes, social support at work does not seem to be an appropriate variable to discriminate between employees at low and high risk in our study population.

In addition to the constraints inherent in classifying job strain, defining casual BP also poses challenges. It is important to classify individuals as hypertensives or normotensives for purposes of practical clinical decision-making, but assessing BP in this binary manner overlooks differences in SBP and DBP. Indeed, these two measures have different hemodynamic significance and are influenced differently by biological, behavioral, and psychosocial determinants. In agreement with our findings, other studies have found more consistent evidence of an association between job strain and SBP than with DBP [29,62]. It is extremely important to understand these differences, as studies have shown that SBP is not only more difficult to control [53] but also a more important cardiovascular risk factor than DBP [63,64].

Our study also established that individuals using antihypertensive drugs should be evaluated separately from other hypertensives, because the relationship between job strain and casual BP differs with the use of such drugs. However, many studies of workplace stress and BP have considered the use of antihypertensive drugs to be a confounding variable [65,66] or opted to exclude medicated hypertensives from their analyses [29,67], thus missing the opportunity to observe the role of antihypertensive drugs in this relationship. The same occurs when BP is defined as a binary outcome in order to avoid the difficulties of including use of medication as a variable in the analyses.

Further studies may adopt a set of strategies to avoid misclassification, including the use of standardized instruments and techniques to measure exposure and outcome variables; the evaluation of BP in a continuous manner, taking use of antihypertensive drugs into account; and the combination of multiple measurements of job strain and BP. Additionally, in studies with a large sample size, the strategy applied here of excluding the participants who were more susceptible to misclassification for the exposure and outcome variables might be useful. However, the criteria used for the exclusion of the participants should be based in a very thorough exploratory analysis, such as the one in this paper.

This study presents findings based on baseline data collected with great methodological rigor and operational quality for ELSA-Brasil, a cohort of civil servants with wide-ranging geographical and population coverage. The slight increase observed in BP levels among the high job strain category is very important at a population level. There is evidence that a reduction of 2 mmHg in population-mean systolic BP may reduce mortality from stroke and cardiovascular disease by 10% and 7%, respectively [22].

One strength of this paper is the large study population, which made it possible to exclude participants who were more susceptible to misclassification for the exposure and outcome variables. We acknowledge that this procedure leads to loss of information and may have partially biased our results. However, our goal was to show that misclassification, one of the most important sources of bias in studies about job strain and high BP [11], leads to a dilution of the differences between comparison groups and might explain some of the inconsistencies encountered in this research field. It must be emphasized also that, as we excluded only those in the second tertile of both dimensions, we still have participants on the border between quadrants on the lower and higher ends of the dimensions. We have adopted this procedure to prevent excessive loss of information and believe that this has not affected our results significantly for two reasons. First, the participants in the central portion of the distribution are the most susceptible to misclassification because it can occur simultaneously in both dimensions. Second, as we later combined the quadrants into two job strain categories (low versus high job strain), we still had participants on the extreme ends of the job control dimension.

Given the constraints of this study, the cross-sectional nature of the analyses precludes establishing a time relation, and possible reverse causality cannot be discarded. In most cases, however, high BP is a silent condition, and its symptoms are hardly likely to modify the status of exposure to job strain. On the other hand, prior awareness of a high BP condition might influence the self-reported status of exposure to job strain. However, as we compared BP levels by job strain category within each subgroup (normotensives, medicated hypertensives, and unmedicated hypertensives), those who knew they were hypertensive (mostly included in the subgroup of medicated hypertensives) were compared to each other. Thus, information bias is improbable. Selection bias, although always possible in studies comprised of voluntary participants, is also improbable due to two features of ELSA-Brasil; the recruitment goals and the recruitment strategies, which did not mention specific risk factors or diseases but simply proposed participation in a long-term health study to contribute to the generation of scientific knowledge. Thus, it is unlikely that participants and non-participants differed simultaneously with regard both job strain and casual BP (i.e., the relationship between the exposure and the outcome is distorted). The ELSA-Brasil cohort does not constitute a representative sample of the general population, and therefore generalization of the results must be made carefully. However, the multicenter nature of the study and the predefined proportions of age groups and occupational categories guaranteed the demographic, social, and regional diversity of the study population. The analytical approach used in this paper did not allow adjustment for potential confounders. Thus, confounding an important source of systematic error cannot be discarded in our study. Our use of casual BP measurement may also represent a limitation. It has been pointed out that work-related stress initially increases BP at work. Alterations in casual BP appear to occur only at a later stage, as a result of structural changes caused by chronic exposure to work stressors [68]. Our results may thus be underestimated, and further studies using ambulatory BP measurements may provide additional information. Lastly, for the small proportion of interviewees who were night workers (5.7%), we have no information as to whether the BP measurements were taken after a night’s work. However, the comprehensive set of measurements carried out at baseline in ELSA-Brasil required lengthy preparation, including 12-h urine collection and overnight fasting (10 to 14 h) before blood collection. This preparation was facilitated by allowing participants to choose their arrival time at the study clinic and, if necessary, to be absent from work the night before the clinical evaluation. We thus believe it unlikely that the acute effects of shift work may have led to an overestimation of the results.

## 5. Conclusions

In conclusion, the findings of this study contribute to the discussion of important issues in the field; the relationship between job strain and casual BP varies across the whole BP distribution. There also are differences according the use of antihypertensive drugs, age, and the BP parameter evaluated (SBP or DBP). Misclassification of the exposure and outcome variables also affects the results. Omitting these issues during data analysis can prevent the identification of important aspects of this relationship.

Considering our results, we recommended a combination of different analytical strategies to address the complex relationship between job strain and BP. In addition to the conventional methods used in this field, analytical approaches that explore the heterogeneity of the association in relation to the whole outcome distribution should be also adopted, such as density curves, quantile-quantile plots, and quantile regression analyses.

## Figures and Tables

**Table 1:** Characteristics of study population. ELSA-Brasil, 2008–2010 (*n* = 9023).

Variables	*n* (%) or Mean (SD)
**Age (years)**	49.3 (7.4)
**Gender (%)**	
Women	4704 (52.1)
Men	4319 (47.9)
**Colour/race (%)**	
White	4589 (51.4)
Brown	2624 (29.4)
Black	1414 (15.9)
Yellow	210 (2.4)
Indigenous	84 (0.9)
**Education (%)**	
<Secondary complete	1018 (11.3)
Secondary complete	3163 (35.0)
Undergraduate complete	1363 (15.1)
Postgraduate	3479 (38.6)
**Job strain scores (units)**	
Job control	17.8 (3.3)
Psychological job demands	13.3 (3.1)
**BP levels (mmHg)**	
SBP	119.5 (16.7)
DBP	76.2 (11.0)
**Use of antihypertensives (%)**	
Yes	2172 (24.1)
No	6851 (75.9)

BP = blood pressure; SD = Standard Deviation.

**Table 2:** Two-sample Kolmogorov-Smirnov (K-S) tests and mean differences (95% CI), considering the three subgroups examined (normotensives, medicated hypertensives, and unmedicated hypertensives), for the total sample and stratified by gender and age. ELSA-Brasil, 2008–2010 (*n* = 9023).

Group	K-S Statistic (*D*) ^1^		Mean Difference ^2^ and 95% CI (mmHg)			
	SBP	DBP	SBP		DBP	
**All (*n* = 9023)**						
Normotensive	0.03	0.03	0.39	(−0.17, 0.94)	0.42 *	(0.02, 0.82)
Medicated hypertensive	0.11 ***	0.07 *	4.30 ***	(2.81, 5.78)	1.52 **	(0.60, 2.45)
Unmedicated hypertensive	0.12 **	0.04	1.47	(−0.44, 3.38)	0.08	(−1.05, 1.21)
**Women (*n* = 4704)**						
Normotensive	0.04	0.06 **	1.01 **	(0.28, 1.73)	0.86 **	(0.33, 1.39)
Medicated hypertensive	0.14 ***	0.11 **	4.44 ***	(2.29, 6.59)	1.25	(−0.07, 2.57)
Unmedicated hypertensive	0.18 *	0.12	0.12	(−3.44, 3.68)	−1.36	(−3.19, 0.47)
**Men (*n* = 4319)**						
Normotensive	0.06 *	0.05	0.96 *	(0.22, 1.70)	0.61 *	(0.04, 1.18)
Medicated hypertensive	0.12 ***	0.11 **	5.13 ***	(3.04, 7.21)	2.44 ***	(1.11, 3.77)
Unmedicated hypertensive	0.12 *	0.08	2.39 *	(0.04, 4.73)	1.05	(−0.41, 2.50)
**<50 years old (*n* = 4731)**						
Normotensive	0.02	0.02	−0.16	(−0.85, 0.53)	0.12	(−0.39, 0.63)
Medicated hypertensive	0.05	0.04	1.00	(−1.39, 3.38)	0.42	(−1.17, 2.00)
Unmedicated hypertensive	0.14	0.05	1.31	(−1.20, 3.82)	0.34	(−1.17, 1.85)
**50 years old or more (*n* = 4292)**						
Normotensive	0.07 **	0.07 *	1.52 **	(0.61, 2.43)	0.95 **	(0.31, 1.60)
Medicated hypertensive	0.16 ***	0.09 **	6.19 ***	(4.32, 8.06)	1.86 **	(0.72, 3.00)
Unmedicated hypertensive	0.15 *	0.08	2.24	(−0.43, 4.91)	−0.27	(−1.91, 1.37)

^1^ The K-S statistic quantifies the highest distance between the empirical distribution functions of casual BP for the two samples (high and low job strain categories); ^2^ Difference between casual BP means of the high and low job strain categories. * *p* ≤ 0.05; ** *p* ≤ 0.01; *** *p* ≤ 0.001. CI: Confidence Interval.
